# Phagocytosis Escape by a *Staphylococcus aureus* Protein That Connects Complement and Coagulation Proteins at the Bacterial Surface

**DOI:** 10.1371/journal.ppat.1003816

**Published:** 2013-12-12

**Authors:** Ya-Ping Ko, Annemarie Kuipers, Claudia M. Freitag, Ilse Jongerius, Eva Medina, Willemien J. van Rooijen, András N. Spaan, Kok P. M. van Kessel, Magnus Höök, Suzan H. M. Rooijakkers

**Affiliations:** 1 Center for Infectious and Inflammatory Disease, Institute of Bioscience and Technology, Texas A&M University Health Science Center, Houston, Texas, United States of America; 2 Medical Microbiology, University Medical Center Utrecht, Utrecht, The Netherlands; 3 Utrecht University, Faculty of Veterinary Medicine, Department of Infectious Diseases and Immunology, Utrecht, The Netherlands; 4 Sir William Dunn School of Pathology, University of Oxford, Oxford, United Kingdom; 5 Infection Immunology Research Group, Helmholtz Centre for Infection Research, Braunschweig, Germany; Vanderbilt University, United States of America

## Abstract

Upon contact with human plasma, bacteria are rapidly recognized by the complement system that labels their surface for uptake and clearance by phagocytic cells. *Staphylococcus aureus* secretes the 16 kD Extracellular fibrinogen binding protein (Efb) that binds two different plasma proteins using separate domains: the Efb N-terminus binds to fibrinogen, while the C-terminus binds complement C3. In this study, we show that Efb blocks phagocytosis of *S. aureus* by human neutrophils. *In vitro*, we demonstrate that Efb blocks phagocytosis in plasma and in human whole blood. Using a mouse peritonitis model we show that Efb effectively blocks phagocytosis *in vivo*, either as a purified protein or when produced endogenously by *S. aureus*. Mutational analysis revealed that Efb requires both its fibrinogen and complement binding residues for phagocytic escape. Using confocal and transmission electron microscopy we show that Efb attracts fibrinogen to the surface of complement-labeled *S. aureus* generating a ‘capsule’-like shield. This thick layer of fibrinogen shields both surface-bound C3b and antibodies from recognition by phagocytic receptors. This information is critical for future vaccination attempts, since opsonizing antibodies may not function in the presence of Efb. Altogether we discover that Efb from *S. aureus* uniquely escapes phagocytosis by forming a bridge between a complement and coagulation protein.

## Author Summary

*Staphylococcus aureus* is a leading cause of severe bacterial infections in both hospital and community settings. Due to its increasing resistance to antibiotics, development of additional therapeutic strategies like vaccination is required to control this pathogen. Vaccination attempts against *S. aureus* have not been successful so far and an important reason may be the pathogen's elaborate repertoire of molecules that dampen the immune response. These evasion molecules not only suppress natural immunity but also hamper the current attempts to create effective vaccines. In this paper, we describe a novel mechanism by which *S. aureus* can prevent uptake by phagocytic immune cells. We discover that the secreted *S. aureus* protein Extracellular fibrinogen binding protein (Efb) generates a ‘capsule’-like shield around the bacterial surface through a dual interaction with the plasma proteins complement C3b and fibrinogen. The Efb-dependent fibrinogen shield masks important opsonic molecules like C3b and antibodies from binding to phagocyte receptors. This information is critical for future vaccination attempts, since opsonizing antibodies may not function in the presence of this anti-phagocytic shield.

## Introduction

Phagocytosis by neutrophils is crucial to the host innate defense against invading bacteria since it leads to intracellular destruction of bacteria by production of oxygen radicals and proteolytic enzymes [1]. Bacterial engulfment by neutrophils is strongly enhanced by the labeling or ‘opsonization’ of bacteria with plasma factors such as antibodies and complement activation products (C3b, iC3b) [2]. Complement activation takes place at the bacterial surface and is initiated by recognition molecules (C1q, Mannose Binding Lectin (MBL)) that interact with bacterial surface structures like sugars or proteins [3]. Complement activation occurs through three different pathways (classical, lectin and alternative) that converge in the formation of C3 convertase enzymes that cleave the central complement protein C3 [4]. This cleavage step leads to massive decoration of the bacterial surface with covalently deposited C3b and iC3b molecules, which are recognized by complement receptor 1 and 3 (CR1 and CR3) on neutrophils. Complement activation proceeds by formation of C5 convertase enzymes that cleave C5 to release the potent chemoattractant C5a and C5b, which initiates formation of the membrane attack complex [5].

*Staphylococcus aureus* is an important human pathogen notorious for its ability to cause both community- and hospital-acquired diseases, ranging from mild skin infections to bacteremia, sepsis and endocarditis [6]. Although Methicillin-resistant *S. aureus* (MRSA) was previously considered as an opportunistic pathogen causing hospital-acquired infections in immune-compromised patients, the emergence of the highly virulent community-associated MRSA showed that this bacterium could also cause serious infections in otherwise healthy persons [7]. Due to the rapid emergence of antibiotic resistance strains, alternative therapy options are now being explored [8]. Vaccination has not been successful so far and an important reason may be the bacteria's elaborate immune evasion repertoire. Therefore, immune evasion proteins are now considered as important vaccination targets [9]. One proposed vaccine candidate is Extracellular fibrinogen binding protein (Efb), a 16-kD secreted protein with a presumable role in disease pathogenesis [10], [11], which is found in 85% of *S. aureus* strains [12]. Efb consists of two functionally distinct domains: a disordered 9 kD N-terminus (Efb-N) that harbors two binding sites for fibrinogen (Fg) [13] and a folded 7 kD C-terminus (Efb-C) that binds to the C3d domain of complement C3 (which is also present in C3b and iC3b) [14], [15]. Although previous papers described various functions for the isolated N- and C-terminal domains of Efb [12]–[19], it is currently not understood why the full-length Efb protein harbors both a Fg and C3d binding site. In this study we demonstrate that Efb potently blocks phagocytosis of bacteria via a novel mechanism linking the complement and coagulation proteins.

## Results

### Full-length Efb inhibits phagocytosis in the presence of plasma

**Figure 1:**
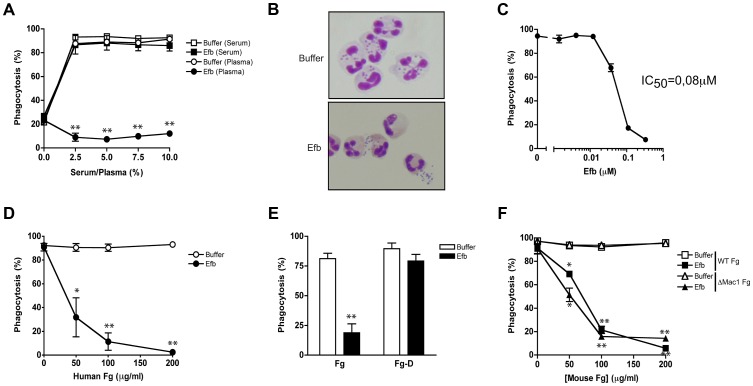
Full-length Efb inhibits phagocytosis of *S. aureus* in human plasma. A. Phagocytosis of fluorescently labeled *S. aureus* by purified human neutrophils in the presence of human serum or plasma and Efb (0.5 µM). B. Histology image of human neutrophils incubated with *S. aureus* and 2.5% plasma in the presence or absence of Efb (0.5 µM). Cells were stained using Diff-Quick. C. Dose-dependent phagocytosis inhibition by Efb in the presence of 2.5% human plasma. IC_50_ was calculated using non-linear regression analysis, R^2^ = 0.95. D–F. Phagocytosis in the presence of 5% human serum supplemented with either full-length human Fg (Fig. 1D), the D domain of human Fg (1 µM or 86 µg/ml) (Fig. 1E) or mouse Fg (WT or lacking the Mac-1 binding site) (Fig. 1F). A, C–F are mean ± se of three independent experiments. B is a representative image. *P<0.05, **P<0.005 for Efb versus buffer (two-tailed Student's *t*-test).

To study a potential role for full-length Efb in phagocyte escape, we mixed fluorescently labeled *S. aureus* with purified human neutrophils, Efb (0.5 µM) and human serum or plasma as a source for complement and analyzed bacterial uptake by flow cytometry. In the presence of serum, Efb did not affect bacterial uptake by neutrophils (Fig. 1A). However when we used human plasma as a complement source, we found that Efb strongly prevented phagocytosis (Fig. 1A,B) and subsequent bacterial killing by neutrophils (Supplemental Fig. S1). Phagocytosis inhibition in plasma occurred in a dose-dependent fashion with a calculated IC_50_ of 0.08 µM (Fig. 1C). Since the main difference between plasma and serum lies in the presence of coagulation proteins, we investigated whether the observed differences in phagocytosis inhibition were caused by the fact that serum lacks Fg. Indeed, we observed that supplementation of serum with physiological concentrations of Fg led to phagocytosis inhibition by Efb (Fig. 1D). Fg is a large (340 kD) dimeric protein that comprises one central E-fragment and two lateral D-fragments. Since Efb binds to the D-fragment of Fg [13], we examined if supplementing serum with Fg-D would also lead to phagocytosis inhibition by Efb. Interestingly, we found that Efb could not block phagocytosis in the presence of Fg-D (Fig. 1E) indicating that full-length Fg is required for phagocytosis inhibition by Efb. Since Fg is a ligand for CR3 (or Mac-1 [20]) on neutrophils, we studied whether the binding of Fg to this receptor is important for the anti-phagocytic effect of Efb. Therefore, we purified Fg from wild-type mice or Fgγ^390–396A^ (ΔMac-1 Fg) mice that express a mutated form of Fg lacking the Mac-1 binding site but retaining clotting function [21]. Fig. 1F shows that supplementation of human serum with both forms of mouse Fg led to inhibition by Efb, indicating that Fg binding to Mac-1 is not important for inhibition. In conclusion, Efb interferes with phagocytosis in a plasma environment and the presence of full-length Fg is required for this inhibition.

### Simultaneous binding to Fg and C3 is essential for phagocytosis inhibition by Efb

**Figure 2:**
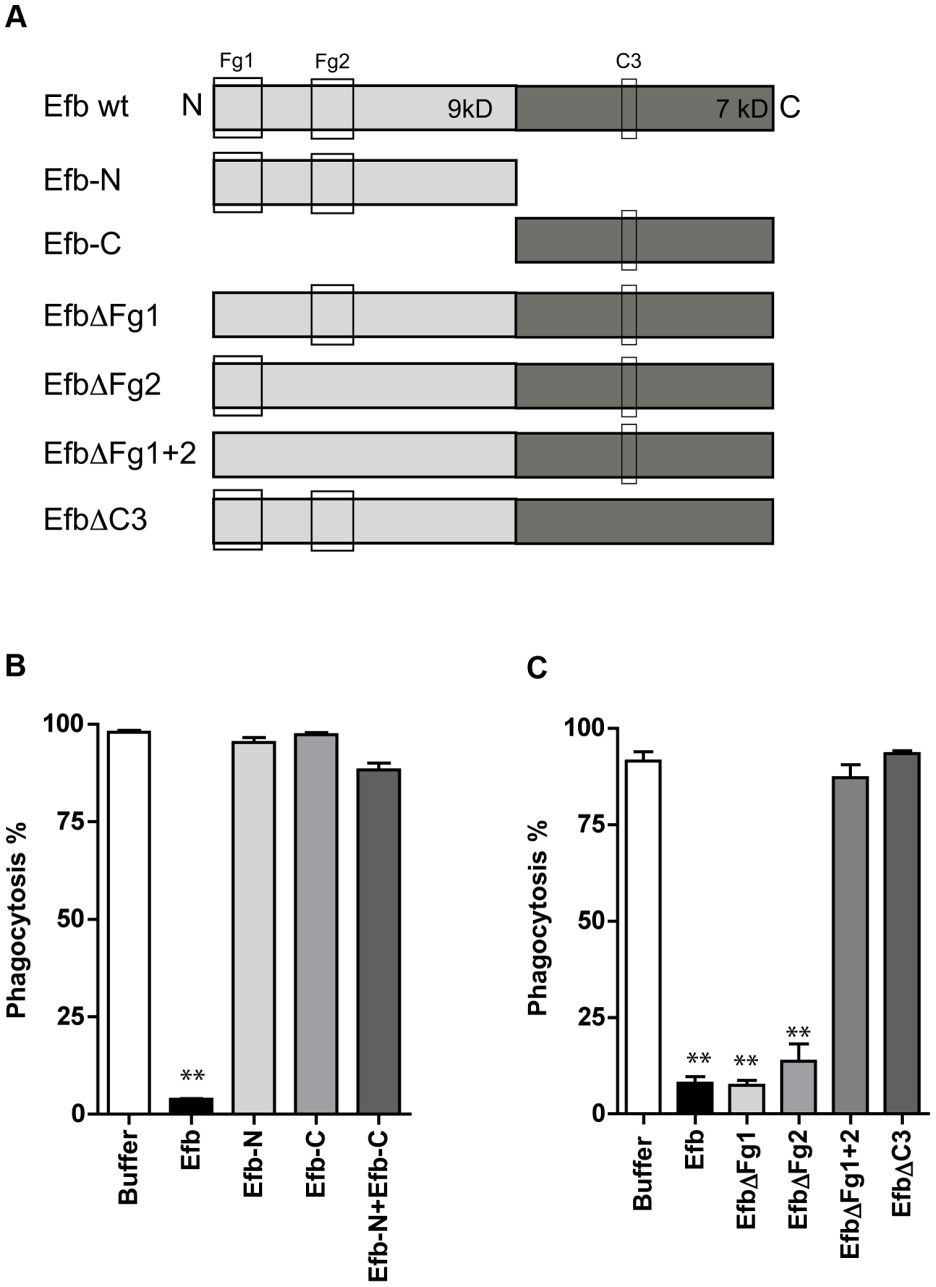
Simultaneous binding to Fg and C3 is essential for phagocytosis inhibition by Efb. A. Schematic overview of Efb mutants generated in this study. Efb is depicted in its secreted form (30–165) lacking the signal peptide (1–29). Bounding boxes indicate Fg- and C3-binding domains. The N-terminus of Efb (light grey, 9 kD) harbors two Fg binding sites named Fg1 (residues 30–67) and Fg2 (residues 68–98). The C-terminus of Efb (dark grey, 7 kD) harbors the C3 binding site (residues R131 and N138). EfbΔFg1 has deletion of residues 30–45, resulting in non-functional binding Fg1; whereas EfbΔFg2 has deletion of residues 68–76, resulting in non-functional binding Fg2. B–C. Phagocytosis of fluorescent *S. aureus* by human neutrophils in the presence of 5% human plasma and Efb fragments (B) or Efb mutants (C) (all at 1 µM). B,C are mean ± se of three independent experiments. **P<0.005 for Efb versus buffer (two-tailed Student's *t*-test).

To get more insight into the mechanism of inhibition, we constructed a panel of Efb mutants (Fig. 2A). First we observed that the individual N- or C-termini of Efb could not block phagocytosis in plasma (Fig. 2B). In addition, mixing the N- and C-terminal fragments of Efb did not markedly affect phagocytosis, indicating that full-length Efb is required. Second, we generated mutants of full-length Efb lacking the previously characterized binding sites for Fg and C3 (Fig. 2A [13], [14]). We created three different Fg-binding mutants: EfbΔFg1 lacking residues 30–45, EfbΔFg2 lacking residues 68–76 and EfbΔFg1+2 lacking both these Fg binding sites. Furthermore we created EfbΔC3 in which the C3d-binding residues R131 and N138 were each replaced with a glutamic acid (E) (also known as Efb-RENE [14]). Using direct binding ELISAs we verified that EfbΔFg1+2 could no longer bind Fg, while the single EfbΔFg1 and EfbΔFg2 mutants and EfbΔC3 still bound Fg (Supplemental Fig. S2A). As expected, all mutants except EfbΔC3 bound to C3b (Supplemental Fig. S2B). Next, we compared these mutants in the neutrophil phagocytosis assay in the presence of human plasma. We show that EfbΔFg1+2 and EfbΔC3 could no longer block phagocytosis (Fig. 2C), indicating that a simultaneous interaction with both Fg and complement C3 (products) is essential for the anti-phagocytic action of Efb. The finding that EfbΔFg1 and EfbΔFg2 were still active indicates that Efb requires only one of its two Fg binding sites to block phagocytosis.

### Efb blocks phagocytosis *ex vivo* and *in vivo*

**Figure 3:**
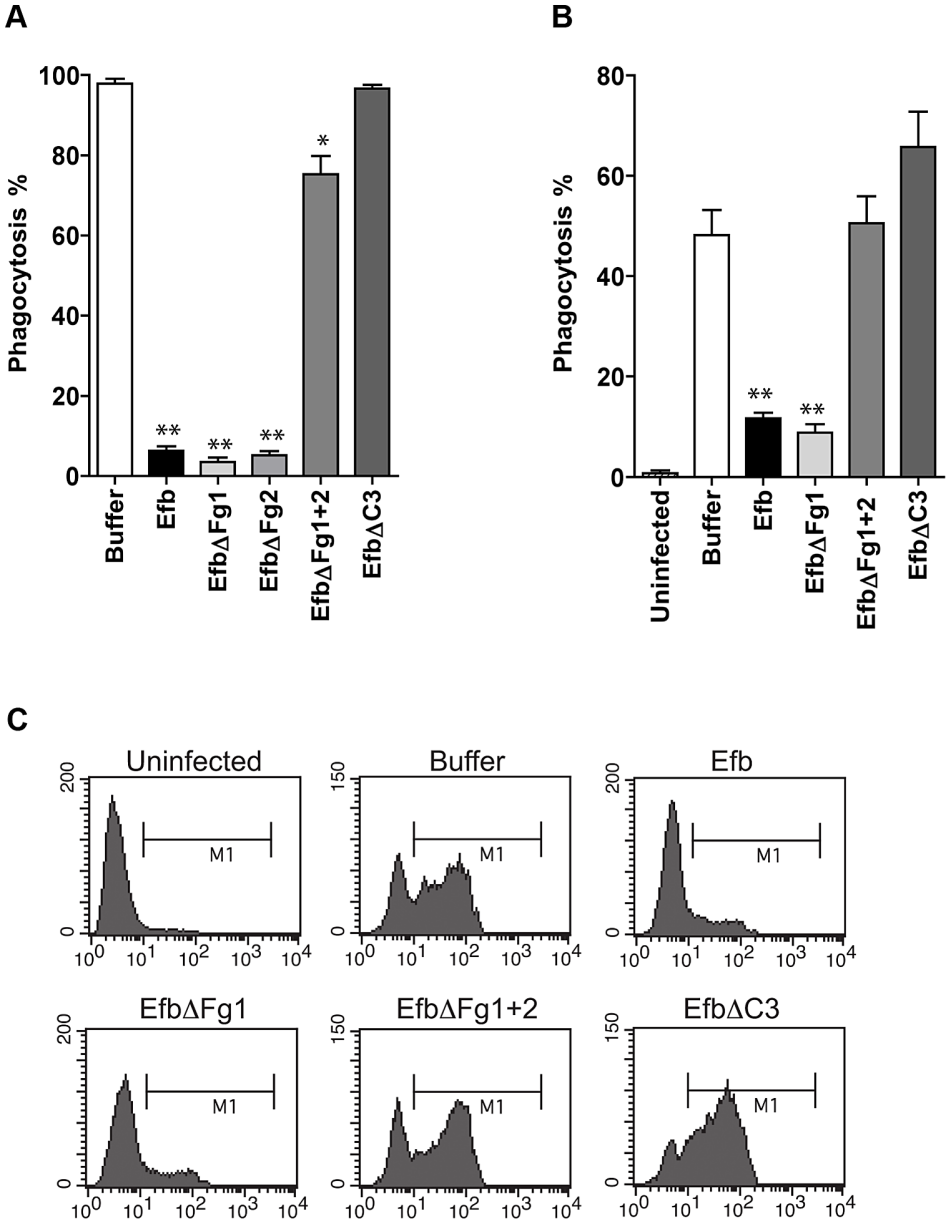
Purified Efb blocks phagocytosis *ex vivo* and *in vivo*. A. *Ex vivo* phagocytosis of fluorescent *S. aureus* incubated with 50% human whole blood and Efb (1 µM). Neutrophils were gated based on forward and side scatter properties. B. *In vivo* phagocytosis of fluorescent *S. aureus* by human neutrophils in the mouse peritoneum. Neutrophils were attracted to the peritoneal cavity using carrageenan (i.p.) and subsequently challenged with 10^8^ heat-inactivated fluorescent *S. aureus* and Efb (1 µM) for 1 hour. The peritoneal lavage was collected and neutrophil phagocytosis was analyzed by flow cytometry. Neutrophils were gated based on Gr-1 expression. The mouse experiments were carried out three times. In each experiment, we used 3 mice per group and the cells of these 3 mice were pooled for phagocytosis analysis. C. Representative histograms of B. A,B are mean ± se of three independent experiments. *P<0.05, **P<0.005 for Efb versus buffer (two-tailed Student's *t*-test).

To study whether Efb can also block phagocytosis in a natural environment, we tested its activity in *ex vivo* and *in vivo* phagocytosis models. In an *ex vivo* human whole blood model, we incubated fluorescent *S. aureus* with 50% human whole blood and Efb. After 25 minutes, neutrophil phagocytosis was analyzed by flow cytometry. We observed that full-length Efb potently blocked phagocytosis by human neutrophils in whole blood (Fig. 3A) and that this inhibition depends on the interaction of Efb with both Fg and C3. Next, we studied phagocytosis of *S. aureus* in an *in vivo* mouse peritonitis model. To this end, mice were treated with carrageenan intraperitoneally (i.p.) to induce neutrophil infiltration into the peritoneal cavity and subsequently challenged with 10^8^ heat-inactivated fluorescent *S. aureus* in the presence or absence of Efb (1 µM). One hour later, mice were sacrificed and the peritoneum was lavaged with sterile PBS. Neutrophils were stained and phagocytosis of fluorescent bacteria was analyzed by flow cytometry. We observed that Efb blocked phagocytosis in the peritoneum (Fig. 3B,C). Efb mutants showed that inhibition of phagocytosis *in vivo* also depends on the Fg and C3 binding domains of Efb.

### Phagocytosis inhibition by Efb is independent of complement inhibition

**Figure 4:**
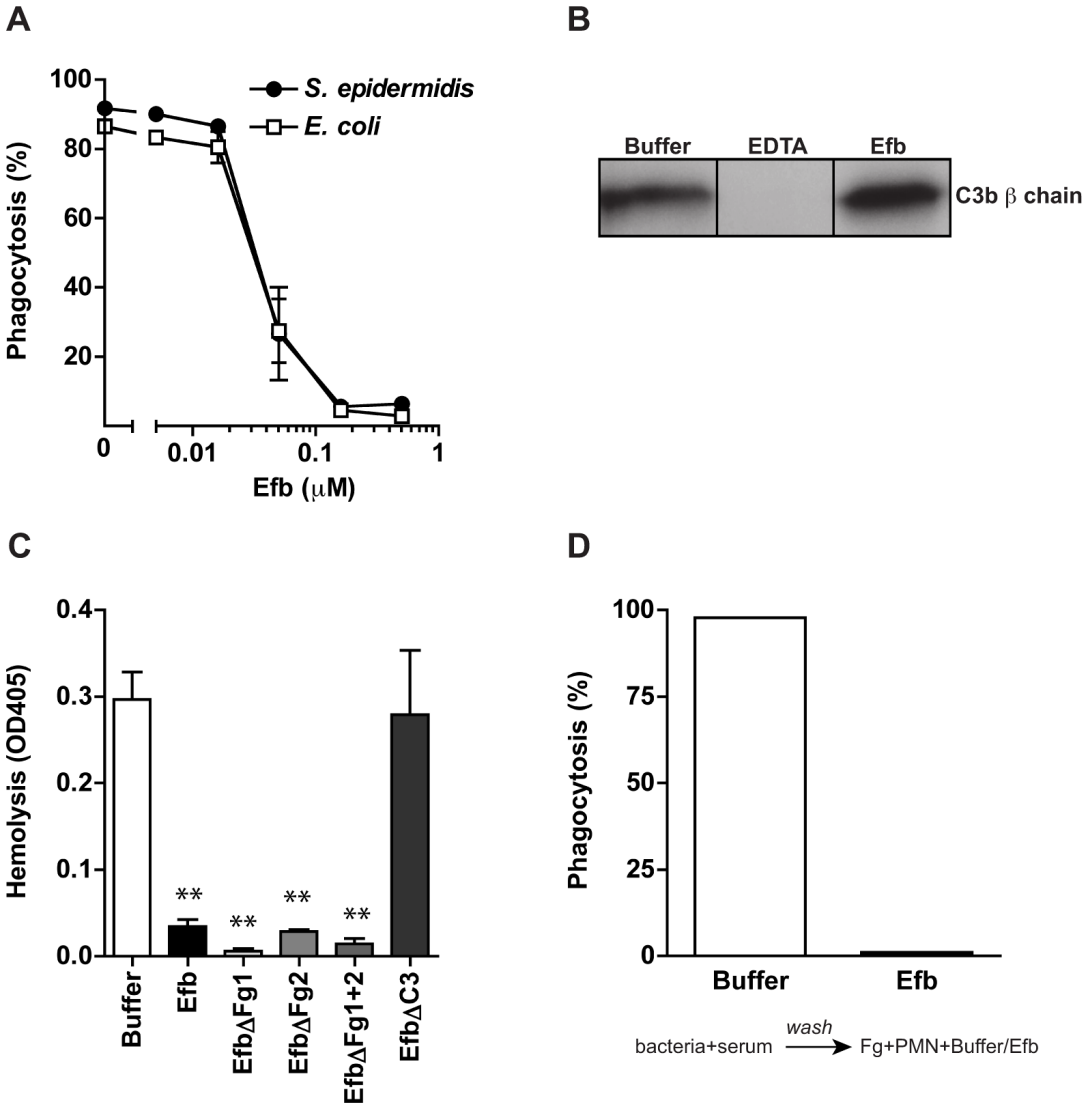
Phagocytosis inhibition by Efb is independent of complement inhibition. A. Phagocytosis of fluorescently labeled *S. epidermidis* and *E. coli* by purified human neutrophils in the presence of human plasma (5%) and Efb. B. Immunoblot detecting surface-bound C3b after incubation of *S. aureus* with 5% human plasma in the presence of 5 mM EDTA or 0.5 µM Efb. Blot is a representative of 3 independent experiments. C. Alternative pathway hemolysis of rabbit erythrocytes in 5% human plasma and Efb (mutants) (1 µM). Bars are the mean ± se of three independent experiments. **P<0.005 for Efb versus buffer (two-tailed Student's *t*-test). D. Phagocytosis with a washing step. Fluorescent *S. aureus* was first incubated with 5% serum to deposit complement. Bacteria were washed and subsequently mixed with neutrophils and Fg in the presence or absence of Efb (0.5 µM). Graph is a representative of three independent experiments.

Experiments shown above indicate that Efb requires an interaction with both complement and Fg to block phagocytosis. To study whether Efb also interacts with *S. aureus* specifically, we analyzed whether purified Efb can block phagocytosis of other bacteria as well. Fluorescent *S. epidermidis* or *E. coli* were mixed with human plasma and phagocytosis by neutrophils was evaluated. Efb potently inhibits the uptake of these bacteria as well, indicating that Efb can block phagocytosis independently of *S. aureus* (Fig. 4A). Previously, we observed that the C-terminal domain of Efb is a complement inhibitor that inactivates C5 convertases to prevent cleavage of C5 [12]. Efb-C did not affect C3b labeling of bacteria in conditions where all complement pathways are active. However, since the effects of Efb on complement inhibition were performed with serum instead of plasma, we wondered whether full-length Efb might affect C3b labeling of bacteria in a plasma environment. Therefore, we incubated *S. aureus* with human plasma and Efb and quantified surface-bound C3b using immunoblotting. As a control, we added EDTA to prevent activation of all complement routes (which are calcium and magnesium dependent). Similar amounts of C3b were found on the bacterial surface in the presence of Efb compared to buffer (Fig. 4B), indicating that Efb does not interfere with C3b labeling in plasma. Subsequently, we re-analyzed the inhibition of C5 convertases by Efb (mutants) in plasma using an alternative pathway hemolytic assay. Rabbit erythrocytes were incubated with human plasma and C5 cleavage was measured by means of C5b-9 dependent lysis of erythrocytes. In conjunction with previous results in serum, we found that all Efb mutants except for EfbΔC3 inhibited C5 cleavage in plasma (Fig. 4C). Since this inhibition exclusively depends on the C-terminal domain (all Fg binding mutants of Efb could still block C5 cleavage), this proves that interference with C5 cleavage is at least not sufficient for phagocytosis inhibition by Efb. To further show that the effects of Efb on complement activation are dispensable for phagocytosis inhibition, we introduced a washing step in our phagocytosis assay. Bacteria were first incubated with serum (in the absence of Efb) to deposit C3b. After washing away unbound serum proteins (including C5a), these pre-opsonized bacteria were incubated with Fg and neutrophils. In this assay, Efb could potently block phagocytosis (Fig. 4D). In conclusion, these results indicate that the anti-phagocytic activity of Efb is not related to its complement-inhibitory effect.

### Efb covers *S. aureus* with a shield of Fg

**Figure 5:**
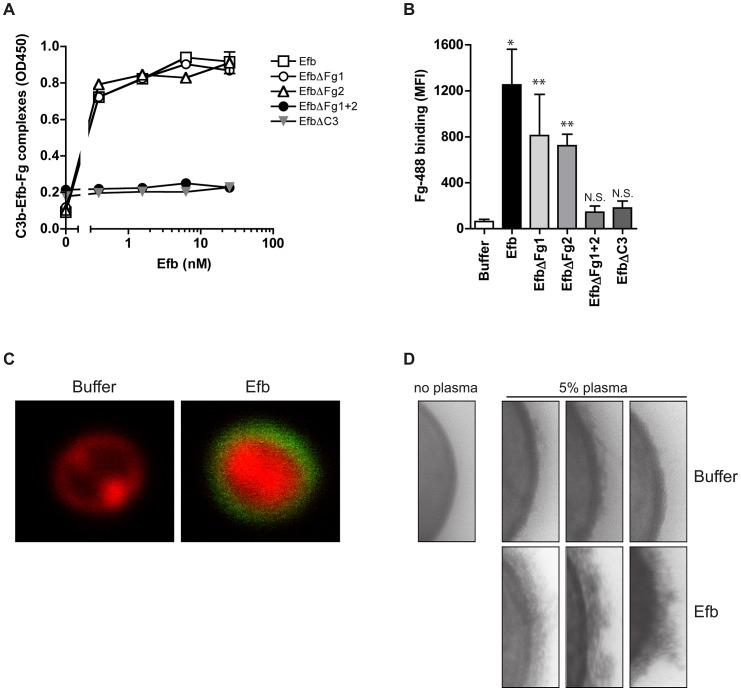
Efb attracts Fg to the bacterial surface. A. ELISA showing that Efb can bind Fg and C3b at the same time. C3b-coated microtiter wells were incubated with Efb (mutants) and, after washing, incubated with 50 nM Fg that was detected with a peroxidase-conjugated anti-Fg antibody (Abcam). Graph is a representative of two independent experiments performed in duplicate. B. Binding of Alexa488-labeled Fg (60 µg/ml) to serum-opsonized *S. aureus* in the presence of Efb (mutants) (0.5 µM). Graph represents mean ± se of three independent experiments. *P<0.05, **P<0.005 for Efb versus buffer (two-tailed Student's *t*-test). N.S. is not significant. C. Confocal analysis of samples generated in B (representative images). D. TEM pictures of *S. aureus* incubated with 5% human plasma in the absence or presence of Efb (0.5 µM). Three representative images are shown.

We wondered whether Efb might bind to C3b-labeled bacteria and then attract Fg to the surface. First, we studied whether full-length Efb can bind to Fg and C3b at the same time. C3b-coated microtiter plates were incubated with Efb and, after a washing step, treated with Fg. Fig. 5A shows that Efb is able to form a complex with C3b and Fg. Also, the EfbΔFg1 and EfbΔFg2 mutants could still form Fg-C3b complexes. In contrast, complex formation was not detected for the mutants that lack either both Fg (EfbΔFg1+2) or the C3 binding domains (EfbΔC3) (Fig. 5A). Then, we investigated whether Efb could attract Fg to pre-opsonized bacteria. Therefore, *S. aureus* was pre-opsonized with human serum to deposit complement and subsequently incubated with Efb. After washing, bacteria were incubated with Alexa-488 conjugated Fg. Using both flow cytometry and confocal microscopy we observed that Efb mediates Fg binding to pre-opsonized bacteria (Fig. 5B,C). Consistent with the ELISA data for complex formation, no Fg binding was detected in the presence of EfbΔFg1+2 or EfbΔC3. Confocal analyses indicated that Efb covers the complete bacterial surface with Fg (Fig. 5C). Using Transmission Electron Microscopy we analyzed this Fg layer created by Efb in more detail. After incubation of *S. aureus* with plasma and Efb, we observed a diffuse outer layer formed around the bacteria (Fig. 5D). Altogether these experiments show that Efb binds to C3b on the bacterial surface and subsequently attracts Fg forming a shield around the bacterial surface.

### Efb blocks recognition of C3b and IgG on the surface

**Figure 6:**
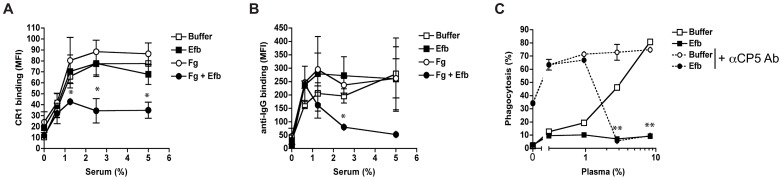
Efb prevents recognition of opsonic C3b and IgG. A–B. Flow cytometry assay detecting binding of soluble CR1 (A) or anti-IgG antibody (B) to pre-opsonized *S. aureus* in the presence of buffer, Efb (0.5 µM) and/or Fg (200 µg/ml). C. Efb inhibits phagocytosis of encapsulated *S. aureus* by human neutrophils. FITC-labeled *S. aureus* strain Reynolds (high capsule CP5 expressing strain) was incubated with human plasma and/or Efb (0.5 µM) in the presence (dotted line) or absence (solid line) of polyclonal rabbit anti-CP5 antibody. All figures represent the mean ± se of three separate experiments. *P<0.05, **P<0.005 for Efb+Fg versus buffer (A,B) or Efb versus buffer (for dotted lines) (two-tailed Student's *t*-test).

Since Efb covers bacteria with a shield of Fg, we hypothesized that this would frustrate the binding of phagocytic receptors to their ligands on the bacterial surface Using flow cytometry, we first analyzed whether C3b-labeled bacteria were still recognized by CR1. Pre-opsonized *S. aureus* was incubated with soluble CR1 in the presence of Fg and Efb. Clearly, binding of CR1 to pre-opsonized bacteria was blocked by the presence of both Fg and Efb (Fig. 6A). Addition of Fg or Efb alone did not affect CR1 binding. Next, we investigated whether the Fg shield specifically blocks C3b-CR1 interactions or whether it also disturbs the binding of neutrophil Fc receptors to opsonic antibodies. To analyze this, we determined whether the Fc part of bacterium-bound IgG could still be recognized by specific antibodies. We found that incubation of pre-opsonized bacteria with Efb and Fg disturbs recognition of the antibody Fc domain on the surface (Fig. 6B), suggesting that Fc receptors can no longer recognize their target. This information is crucial for future vaccine development since opsonic antibodies against *S. aureus* may not function when Efb hides these antibodies underneath an Fg shield. To further prove that Efb functionally blocks opsonization, we analyzed phagocytosis of an encapsulated *S. aureus* strain in the presence or absence of anti-capsular antibodies. The encapsulated *S. aureus* strain Reynolds was grown for 24 hours in Columbia agar supplemented with 2% NaCl (for optimal capsule expression [22]) and subsequently labeled with FITC. We verified capsule expression after FITC-labeling using specific antibodies (Supplemental Fig. S3). In low plasma concentrations (0–1%), we observed that anti-capsular antibodies caused a 6-fold increase in phagocytic uptake of encapsulated *S. aureus* (Fig. 6C). At these plasma concentrations, Efb could not block phagocytosis. However at higher plasma concentrations (3% and more), Efb potently impeded phagocytosis in the presence of anti-capsule antibody (Fig. 6C). These data support our idea that the Fg shield created by Efb prevents recognition of important opsonins like C3b and IgG, also in the context of a capsule-expressing strain that is targeted by specific antibodies.

### Endogenous Efb blocks phagocytosis *in vitro* and *in vivo*

**Figure 7:**
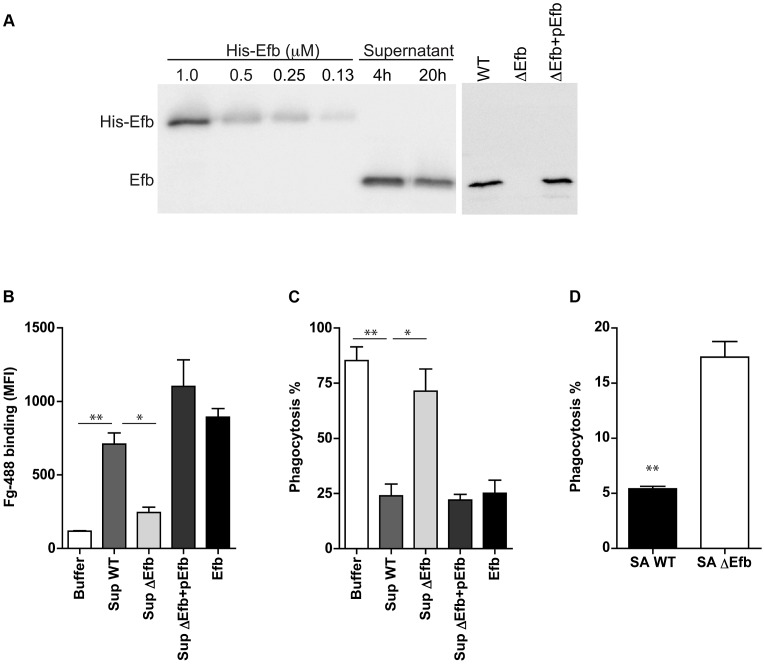
Endogenously produced Efb blocks phagocytosis via complex formation. A. *Left*. Immunoblot detecting Efb in 4 h and 20 h culture supernatants of *S. aureus* Newman; fixed concentrations of His-tagged Efb were loaded as controls. *Right*. Immunoblot of 4 h culture supernatants of *S. aureus* Newman (WT), an isogenic Efb deletion mutant (ΔEfb) and its complemented strain (ΔEfb+pEfb). Blots were developed using polyclonal sheep anti-Efb and Peroxidase-labeled donkey anti-sheep antibodies. Blot is a representative of two independent experiments. B. Flow cytometry analysis of the binding of Alexa488-labeled Fg to pre-opsonized *S. aureus* in the presence of 4 h culture supernatants (2-fold diluted) or purified Efb (250 nM). C. *In vitro* phagocytosis of fluorescently labeled *S. aureus* by purified human neutrophils. Pre-opsonized *S. aureus* was first incubated with 4 h culture supernatants (2-fold diluted) or purified Efb (250 nM) and subsequently mixed with Fg and neutrophils. D. *In vivo* phagocytosis of GFP-expressing wild-type or Efb-deficient *S. aureus* strains by neutrophils in the mouse peritoneal cavity. Neutrophils were attracted to the peritoneal cavity using carrageenan (i.p.) and subsequently injected with 300 µl of GFP-expressing wild-type (SA WT) or Efb-deficient (SAΔEfb) *S. aureus* strains during the exponential phase of growth. The peritoneal lavage was collected 1 h thereafter and neutrophil phagocytosis was analyzed by flow cytometry. Neutrophils were gated based on Gr-1 expression. Graphs in B–D represent mean ± se of three independent experiments. *P<0.05, **P<0.005 for WT versus Buffer or ΔEfb (two-tailed Student's *t*-test).

To study whether endogenous expression of Efb leads to impaired phagocytosis of *S. aureus* via complex formation, we extended our analyses with (supernatants of) an isogenic Efb-deletion mutant in *S. aureus* Newman (previously described in [23]). First we performed Immunoblotting to semi-quantify the production levels of Efb in liquid bacterial culture supernatants. Supernatants of wild-type (WT) *S. aureus* Newman were subjected to Immunoblotting and developed using polyclonal anti-Efb antibodies (Fig. 7A). Efb expression in the supernatant was quantified using ImageJ software and compared with fixed concentrations of purified (His-tagged) Efb using linear regression analysis (R^2^ = 0.986). Efb levels in 4 h and 20 h supernatants contained 1,1 µM and 0,9 µM Efb respectively. Although the Efb levels in strain Newman are suspected to be higher than in other *S. aureus* strains (up to 10-fold [24], due to a point mutation in the SaeR/S regulatory system that drives expression of immune evasion genes [25]), the fact that these levels are >10 times higher than the calculated IC_50_ needed for phagocytosis inhibition (0.08 µM, Fig. 1C), suggests that Efb concentrations required for phagocytosis inhibition can be reached *in vivo*. In a separate Immunoblot, we checked for the presence of Efb in 4 h supernatants of the WT, Efb-deficient (ΔEfb) and the complemented strain (ΔEfb+pEfb) confirming the lack of Efb expression in the mutant (Fig. 7A). Next we used these supernatants to study whether endogenous Efb can mediate C3b-Fg complex formation on the bacterial surface. *S. aureus* was first incubated with serum to deposit C3b, then mixed with bacterial supernatants and subsequently incubated with fluorescently labeled Fg. Whereas WT supernatants attracted Fg to the surface of pre-opsonized bacteria, Efb-deficient supernatants did not mediate complex formation (Fig. 7B). This phenotype was restored in the complemented strain. Then we studied whether endogenous Efb could inhibit phagocytosis by neutrophils *in vitro*. Therefore we repeated the latter experiment (but using fluorescent bacteria and unlabeled Fg) and subsequently mixed the bacteria with human neutrophils. We found that supernatants of WT and complemented strains inhibited phagocytosis, while Efb-deficient supernatants did not influence this process (Fig. 7B). To mimic bacterial phagocytosis during a natural infection, carrageenan-treated mice were injected i.p. with GFP-expressing WT *S. aureus* or the Efb-deficient mutant in their original broth culture and sacrificed 1 h thereafter. Mice were subjected to peritoneal lavage and the percentage of neutrophils with internalized staphylococci was determined by flow cytometry. As depicted in Fig. 7D, the Efb-deficient *S. aureus* strain was phagocytosed by neutrophils to a significantly higher extent than the WT strain despite of the fact that the amount of inoculated bacteria was comparable in both groups (app. 2×10^7^). These observations demonstrate that the levels of Efb produced by *S. aureus* are sufficient for preventing phagocytosis *in vivo*.

## Discussion

**Figure 8:**
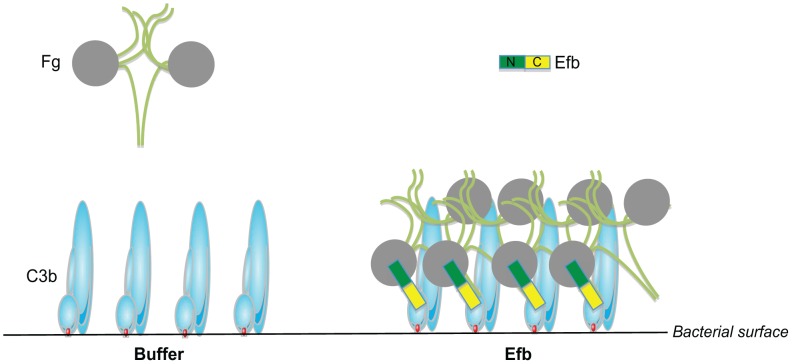
Proposed mechanism for phagocytosis inhibition by Efb. Schematic picture of the proposed phagocytosis escape mechanism by Efb. *Left*, Complement activation on the bacterial surface results in massive labeling of *S. aureus* with C3b molecules while Fg stays in solution. *Right*, *S. aureus* secretes Efb, which binds to surface-bound C3b via its C-terminal domain (colored yellow). Using its N-terminus (green), Efb attracts Fg to the bacterial surface. This way, *S. aureus* is covered with a shield of Fg that prevents binding of phagocytic receptors to important opsonins like C3b and IgG.

The coagulation system has a dual role in the host defense against bacterial infections. On one hand, coagulation supports innate defenses by entrapment and killing of invading bacteria inside clots [26] or via the formation of small antibacterial and pro-inflammatory peptides [27], [28]. On the other hand, bacterial pathogens can utilize coagulation proteins to protect themselves from immune defenses. In this study, we find that *S. aureus* effectively protects itself from immune recognition by secreting Efb that specifically attracts Fg from the solution to the bacterial surface creating a capsule-like shield (Figure 8). To accomplish this, Efb forms a multi-molecular complex of soluble Fg and surface-bound C3b. The fact that the levels of C3b at the bacterial surface are high [29] and that Fg is an abundant plasma protein (1.5–4.0 g/L) makes this a very efficient anti-phagocytic mechanism. The Fg shield created by Efb effectively protects *S. aureus* from recognition by phagocyte receptors. We found that the attracted Fg does not only block the binding of C3b to its receptor, but also hides the important opsonin IgG underneath the Fg shield. We think that this information is critical for future vaccine development against *S. aureus*. Generation of protective ‘opsonizing’ antibodies recognizing *S. aureus* surface structures is considered to be an important goal of vaccination. However, these antibodies will not function if they are protected underneath a layer of Fg. We anticipate that including Efb in future vaccines might be beneficial as it could prevent formation of this anti-phagocytic shield and enhance the function of opsonizing antibodies. The fact that Efb is conserved among *S. aureus* strains may make it a suitable vaccine candidate [30].

Next to Efb, *S. aureus* secretes two other proteins that specifically interact with the coagulation system: the *S. aureus* ‘coagulases’ named Coagulase and Von Willebrand factor binding protein are secreted proteins that activate prothrombin in a nonproteolytic manner and subsequently convert Fg into fibrin [31]. Thereby, coagulases embed bacteria within a network of fibrin, protecting them from immune recognition and facilitate formation of *S. aureus* abscesses and persistence in host tissues [32]. Coagulase and Efb are expressed at the same time during infection since they are both regulated by the SaeR/S regulator for secreted (immune evasion) proteins [25], [33]. Based on our study, we hypothesize that Efb may be highly important for proper functioning of Coagulase since Efb can attract Fg to the bacterial surface. This way, Efb may aid Coagulase-dependent fibrin formation to occur close to the bacterial surface instead of in solution. Nevertheless our studies also indicate that Efb can block phagocytosis in the absence of prothrombin and Coagulase. However, in a more complex environment the anti-phagocytic mechanisms of Efb and *S. aureus* Coagulase might work synergistically. Furthermore, it seems tempting to speculate that the ability of Efb to attract Fg to the bacterial surface is also beneficial in other infection processes like adhesion. Since Fg is an important constituent of the extracellular matrix (ECM), Efb might also facilitate binding of C3b-opsonized bacteria to the ECM. In fact, Efb was previously classified as an adhesion molecule belonging to the group of SERAMs (secreted expanded repertoire adhesive molecules) [34]. However, as a secreted protein, Efb cannot facilitate bacterial adhesion if it solely binds to Fg in the ECM without interacting with the bacterial surface. Binding to C3b-labeled bacteria via the Efb C-terminus might therefore be crucial for effective bacterial adhesion to Fg.

The pathogenic potential of *S. aureus* is a result of its versatile interactions with multiple host factors, evidenced by the fact that it can survive at multiple sites of the body causing a wide range of infections. At most body sites, *S. aureus* has to deal with cellular and humoral components of the immune system. However, increasing evidence now suggests that *S. aureus* protects itself from immune defense by forming abscess communities surrounded by capsule-like structures that prevent neutrophil invasion [35], [36]. Our study implicates that Efb might be crucial in the formation of these capsules. Furthermore, our whole blood assays shows that Efb may also play an important role in *S. aureus* survival in the blood allowing it to spread to other sites of the body. Previous studies using animal models have highlighted the critical role of Efb in *S. aureus* pathogenesis. For instance, Efb delays wound healing in a rat wound infection model [10] and is important for *S. aureus* pneumonia and abscess formation in kidneys [23]. Our *in vivo* studies corroborate the *in vitro* findings and suggest that complex formation can occur under physiological conditions *in vivo*. However, the available mouse models do not closely mimic this process during clinical infections in humans. Efb is produced in later stages of bacterial growth, thus the bacteria need time to produce Efb before they come into contact with neutrophils. Since neutrophils need to be recruited from the blood to the site of the infection, there normally is time for Efb production and complex formation, especially in the human host where an infection starts with a low number of bacteria. In contrast, in available mouse models the timing is much different as a high inoculum (up to 10^8^ bacteria) is required to establish an infection and these high numbers of bacteria trigger a strong inflammatory response resulting in that the bacteria are already phagocytized before Efb is produced. For this reason, we have mixed the bacteria with their supernatants to ensure the presence of endogenous Efb during the course of the experiments and have chosen a model in which neutrophils are already attracted to the infection site to focus on the anti-phagocytic activity of the molecule. Future studies are needed to design and execute appropriate animal studies that overcome the limitations of current models and better reflect the clinical situation.

In summary, we describe that full-length Efb can inhibit phagocytosis in a unique way through its dual interaction with complement and Fg. Our study indicates that Efb is a highly effective immune escape molecule that blocks phagocytosis of *S. aureus in vivo*.

## Materials and Methods

### Ethics statement

Study participants provided written informed consent in accordance with the Declaration of Helsinki. Approval was obtained from the medical ethics committee of the UMC Utrecht. Animal experiments were performed in strict accordance with the German regulations of the Society for Laboratory Animal Science (GV-SOLAS) and the European Health Law of the Federation of Laboratory Animal Science Associations (FELASA). All experiments were approved by the ethical board Niedersächsisches Landesamt für Verbraucherschutz und Lebensmittelsicherheit, Oldenburg in Germany (Permit No. 33.9-42502-04-10/0296).

### Bacterial strains, fluorescent labeling and supernatants

In this study we used the laboratory *S. aureus* strains Newman, SH1000, Reynolds and Wood 46 (with low expression of Protein A). The *S. aureus* strain KV27 and the *S. epidermidis* and *E. coli* strains were clinical isolates obtained within the UMCU. Targeted deletion (and complementation) of Efb in *S. aureus* Newman was described previously in [23]. All strains were cultured overnight on Tryptic Soy Blood Agar (BD) or Todd Hewitt Agar (with appropriate antibiotics) at 37°C. The capsule-expressing *S. aureus* strain Reynolds and its isogenic CP5-deficient mutant were a kind gift from Jean Lee (Harvard Medical School, Boston, USA) [22]. To optimize capsule expression, strain Reynolds was grown on Columbia Agar supplemented with 2% NaCl (CSA) for 24 h at 37°C. For fluorescent labeling of strains, bacteria were resuspended in PBS and incubated with 0.5 mg/ml FITC (Sigma) for 30 minutes on ice. Bacteria were washed twice with PBS, resuspended in RPMI medium with HSA and stored at −20°C until further use. For *in vivo* experiments, *S. aureus* Newman and the Efb mutant were transformed with the pCM29 plasmid (kindly provided by Alexander Horswill, University of Iowa) allowing constitutive expression of the superfolder green fluorescent protein (sGFP) via the sarAP1 promoter [37]. To isolate bacterial supernatants, WT and mutant strains were cultured overnight in Todd Hewitt Broth (THB) without antibiotics and subsequently sub cultured in fresh THB for 4 h or 20 h. Cultures were centrifuged at 13,000 rpm and collected supernatants were stored at −20°C until further use.

### Protein expression and purification

Recombinant Efb proteins were generated in *E. coli* as described previously [12], [13]. Briefly, (parts of) the *efb* gene from *S. aureus* strain Newman (without the signal peptide) were amplified by PCR and ligated into either the pGEX-5x-1 vector (GE healthcare) or the pRSETB vector (Invitrogen) for N-terminal fusions with glutathione S-transferase (GST) or polyhistidine respectively. Mutations of the Fg and C3 binding domains were introduced in pGEX plasmids containing full-length GST-Efb as described previously [13]. Recombinant proteins were expressed and purified according to the manufacturer's manual. In all experiments where wild-type Efb was compared with mutants, we used GST-tagged Efb. Otherwise His-tagged Efb was used.

### ELISA

Microtiter plates were coated with human C3b [38] or Fg, blocked with 3% BSA-PBS, and incubated with 6 nM Efb for one hour at room temperature. Efb binding was detected using peroxidase-conjugated rabbit anti-GST polyclonal antibodies (Abcam) and quantified using *0*-phenylenediamine dihydrochloride (Sigma). To study formation of C3b-Efb-Fg complexes, C3b-coated plates were incubated with Efb for one hour at room temperature. After washing, human Fg (50 nM) was added and detected through incubation with peroxidase-conjugated anti-Fg antibodies (Abcam).

### Preparation of Fg-D fragments

D fragments of Fg were generated by digestion of human Fg (Enzyme research) with plasmin (Enzyme research, 10 µg/15 mg Fg) in TBS containing 10 mM CaCl_2_ for 4 hours at 37°C as described earlier [39] with modifications. D fragments (85 kD) were purified by gel filtration on Sephacryl S-200 (GE Healthcare) and analyzed by SDS-PAGE.

### Purification of human blood products

For preparation of plasma, venous blood from 10 healthy volunteers was collected in glass vacutainers (BD) containing the anticoagulant lepirudin (50 µg/ml) [40]. To prepare serum, blood was collected in glass vacutainers (BD) without anticoagulant and allowed to clot for 15 min at room temperature. Plasma and serum were collected after centrifugation for 10 minutes at 4000 rpm at 4°C, pooled and subsequently stored at −80°C. Complement-inactivated serum was prepared by incubation of serum for 30 min at 56°C. Human neutrophils were isolated freshly from heparinized blood using the Ficoll-Histopaque gradient method [41] and used on the same day.

### Mice

C57BL/6 female mice were purchased from Harlan-Winkelmann (Borchen, Germany) and used in experiments when they were between 8 and 10 weeks of age. They were housed in micro isolator cages and given food and water ad libitum.

### Phagocytosis assays

#### Whole blood phagocytosis

FITC-labeled *S. aureus* KV27 (1×10^8^/ml) was incubated with freshly isolated human lepirudin blood (50%) and buffer or Efb (0.5 µM) in RPMI-0.05% HSA for 25 min at 37°C. The reaction was stopped using FACS lysing solution (BD Biosciences); samples were washed with RPMI-0.05% HSA and analyzed by flow cytometry using a FACSCalibur (BD). Gating of cells occurred on basis of forward and side scatter; for each sample we measured the fluorescence intensity of 10,000 gated neutrophils. Phagocytosis was expressed as the percentage of neutrophils that became fluorescent.

#### Phagocytosis with purified neutrophils and plasma/serum

FITC-labeled bacteria (5×10^7^/ml) were mixed with human serum or plasma for 2 min at 37°C in the presence or absence of Efb. Freshly isolated neutrophils (5×10^6^/ml) were added and phagocytosis was allowed for 15 min at 37°C. The reaction was stopped by formaldehyde fixation and analyzed by flow cytometry. Alternatively, phagocytosis mixtures were cytospinned on glass slides and stained using Giemsa-based Diff-Quick solution. To analyze killing, phagocytosis mixtures were not fixed but incubated for an additional 90 minutes before they were diluted into ice-cold water (pH 11) and incubated for 15 min on ice to enable neutrophil lysis. Viable bacteria were quantified by colony enumeration. For Fg supplementation, 5% serum was supplemented with 50–200 µg/ml human or mouse Fg (kindly provided by Dr. Jay L. Degen; purified from plasma of wild type and Fgγ^390–396A^ mice [21]). To analyze the influence of bacterial supernatants on phagocytosis, FITC-labeled *S. aureus* KV27 (2.5×10^7^ cfu) was pre-incubated with human serum for 30 min at 37°C in Veronal Buffered Saline containing Ca^2+^ and Mg^2+^ (VBS^++^). After washing in VBS^++^-0.5% BSA, bacteria were incubated with (2-fold) diluted culture supernatants or purified Efb (250 nM) for 1 hour at 37°C. After washing, bacteria were incubated with purified Fg (60 µg/ml, Invitrogen) in RPMI-HSA for 1 hour at 37°C and subsequently, neutrophils were added (7.5×10^5^ cells) and phagocytosis was allowed for 30 min at 37°C.

#### *In vivo* phagocytosis

*S. aureus* strain SH1000 [42] was grown to mid-log phase, heat-inactivated for 60 min at 90°C, and fluorescently labeled with carboxyfluorescein (Molecular Probes, Göttingen, Germany). To induce infiltration of neutrophils within the peritoneal cavity, mice were treated i.p. with 1 mg of carrageenan (Type IV1, Sigma) 4 and 2 days prior to bacterial challenge. Subsequently, mice were injected i.p. with 200 µl of a solution containing 10^8^ heat-inactivated carboxyfluorescein-labeled *S. aureus* SH1000 and Efb (1 µM). To compare WT and ΔEfb strains, mice were directly inoculated in the peritoneal cavity with 300 µl of GFP-expressing WT or ΔEfb *S. aureus* cultures grown to a late exponential phase. Mice were sacrificed 1 h thereafter, and their peritoneum was lavaged with sterile PBS. Lavage samples were centrifuged and pelleted cells were incubated with purified anti-CD32 antibodies to block the FcR, followed by PE-conjugated anti-mouse Gr-1 antibodies. Cells were washed and quenched with trypan blue (2 mg/ml). Samples were immediately subjected to flow-cytometric analysis using a FACScan (Becton Dickinson, San Jose, California). Neutrophils were gated according to their expression of Gr-1 antigen (FL2). Phagocytosis was expressed as the percentage of neutrophils that became fluorescent.

### Alternative pathway hemolysis assay

Human serum (5%) was incubated with buffer or Efb proteins (1 µM) in HEPES-MgEGTA (20 mM HEPES, 5 mM MgCl_2_, 10 mM EGTA) for 15 min at RT. Rabbit erythrocytes (Biotrading Benelux B.V.) were added and incubated for 60 min at 37°C. Mixtures were centrifuged and hemolysis was determined by measuring the absorbance of supernatants at 405 nm.

### Immunoblotting

To analyze C3b deposition on the bacterial surface, *S. aureus* strain Wood46 (3×10^8^/ml) was incubated with 5% human plasma in the presence of Efb (0.5 µM), EDTA (5 mM) or buffer (HEPES^++^; 20 nM HEPES, 5 mM CaCl_2_, 2.5 mM MgCl_2_, pH 7.4) for 30 min at 37°C shaking at 1100 rpm. Bacteria were washed twice with PBS-0.1% BSA and boiled in Laemmli sample buffer containing Dithiothreitol. Samples were subjected to SDS-PAGE and subsequently transferred to a nitrocellulose membrane (Millipore). C3b was detected using a peroxidase-labeled polyclonal anti-human C3 antibody (Protos Immunoresearch, Burlingame, USA) and developed using Enhanced Chemiluminescence (ECL, GE). To quantify Efb in bacterial supernatants, His-Efb and supernatants were run together on an SDS-PAGE gel. After transfer, blots were developed using a polyclonal sheep anti-Efb antibody (kindly provided by Prof JI Flock), peroxidase-labeled donkey anti-sheep antibodies (Fluka Analytical) and ECL. Bands were quantified using ImageJ software and linear regression analysis was performed using GraphPad software.

### Flow cytometry assays with *S. aureus*

*S. aureus* strain Wood46 (3×10^8^/ml) was pre-incubated with human serum for 30 min at 37°C in VBS^++^ buffer, washed with VBS^++^-0.5% BSA and incubated with Efb (0.5 µM) or 2-fold diluted culture supernatants for 1 hour at 37°C shaking. After another washing step, bacteria were incubated with Alexa-488 conjugated Fg (60 µg/ml, Invitrogen) for 1 hour at 37°C shaking. Washed bacteria were analyzed by flow cytometry using a FACSCalibur (BD). Bacteria were gated on the basis of forward and side scatter properties and fluorescence of 10,000 bacteria was analyzed. Alternatively, we incubated pre-opsonized bacteria with Efb (0.5 µM) and/or unlabeled Fg (200 µg/ml) for 1 hour at 37°C shaking. Washed bacteria were incubated with soluble rCR1 (10 µg/ml, kindly provided by Prof. Atkinson, Washington University, St. Louis, MO), FITC-labeled F(ab′)_2_ anti-human C3 antibody (Protos Immunoresearch) or anti-human IgG antibody for 30 min at 37°C. CR1 was detected using PE-labeled anti-CD35 antibodies (BD Pharmingen); the IgG antibody was detected using goat-anti-mouse PE antibodies (BD Pharmingen). Capsule expression on strain Reynolds was analyzed by incubating bacteria with polyclonal anti-CP5 rabbit serum and Phycoerythrin (PE)-conjugated goat anti-rabbit antibody.

### Confocal microscopy

Samples were transferred to glass slides and air-dried. Membrane dye FM 5-95 (Invitrogen) was added and slides were covered with a coverslip. Confocal images were obtained using a Leica TCS SP5 inverted microscope equipped with a HCX PL APO 406/0.85 objective (Leica Microsystems, The Netherlands).

### Transmission electron microscopy

*S. aureus* strain Wood 46 (3×10^8^) was incubated with human plasma (10%) in the presence or absence of Efb (0.5 µM) in HEPES^++^ for 30 min at 37°C, washed once with PBS-1% BSA and adsorbed to 100 mesh hexagonal Formvar-carbon coated copper grids (Stork-Veco, Zoetermeer, NL). Samples were contrasted with 0.4% uranyl acetate (pH 4.0) and 1.8% methylcellulose [43] and analyzed in a JEOL 1010 transmission electron microscope (JEOL Europe, Nieuw Vennep, the Netherlands) at 80 kV.

### Statistics

Statistical analyses were performed using GraphPad Prism 4.0 package and the differences between groups were analyzed for significance using the two-tailed Student's *t*-test.

### Accession number

The accession number for Efb in *S. aureus* Newman is YP_001332103, locus NWMN_1069.

## Supporting Information

Figure S1 **Purified Efb inhibits killing of** ***S. aureus*** **in human plasma.** Killing of *S. aureus* by purified human neutrophils in the presence of 5% human plasma and Efb (0.15 µM). Data are mean ± se of two independent experiments. *P<0.05 for Efb versus buffer (two-tailed Student's *t*-test).
(PDF)

Figure S2 **Fg- and C3b-binding characteristics of Efb mutants.** ELISA experiments analyzing binding of Efb (mutants) to Fg (A) or human C3b (B). Microtiter wells, coated with 0.25 µg human Fg (A) or C3b (B), were incubated with Efb or Efb mutants (both at 6 nM). Efb binding was detected using peroxidase-conjugated rabbit anti-GST antibodies (Abcam). Data are mean ± se of two independent experiments.
(PDF)

Figure S3 **Capsule expression.** (FITC-labeled) *S. aureus* strain Reynolds and its isogenic CP5-deficient mutant were incubated with Rabbit-anti-CP5 antibodies and PE-labeled goat-anti-rabbit antibodies. Antibody binding was quantified using flow cytometry. Data are mean ± se of two independent experiments.
(PDF)
