# Supplementary figures and images for: Phagocytosis Escape by a Staphylococcus aureus Protein That Connects Complement and Coagulation Proteins at the Bacterial Surface
Source: PLoS Pathog. 2013 Dec 12;9(12):e1003816. doi: 10.1371/journal.ppat.1003816 (PMC3861539; doi:10.1371/journal.ppat.1003816)

Supplemental Figure S1

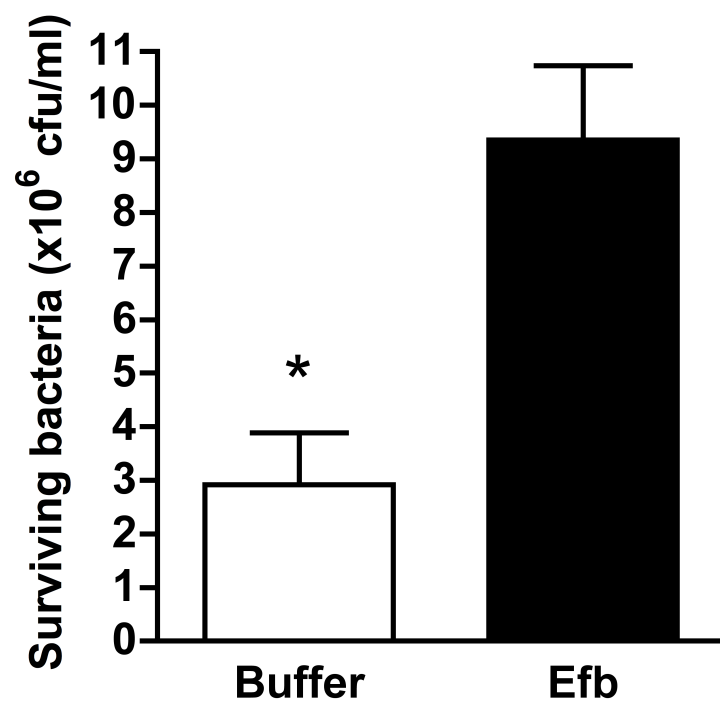

Supplement: Figure S1 — Purified Efb inhibits killing of S. aureus in human plasma. Killing of S. aureus by purified human neutrophils in the presence of 5% human plasma and Efb (0.15 µM). Data are mean ± se of two independent experiments. *P<0.05 for Efb versus buffer (two-tailed Student's t-test). (PDF) [file ppat.1003816.s001.pdf]

Supplemental figure S2

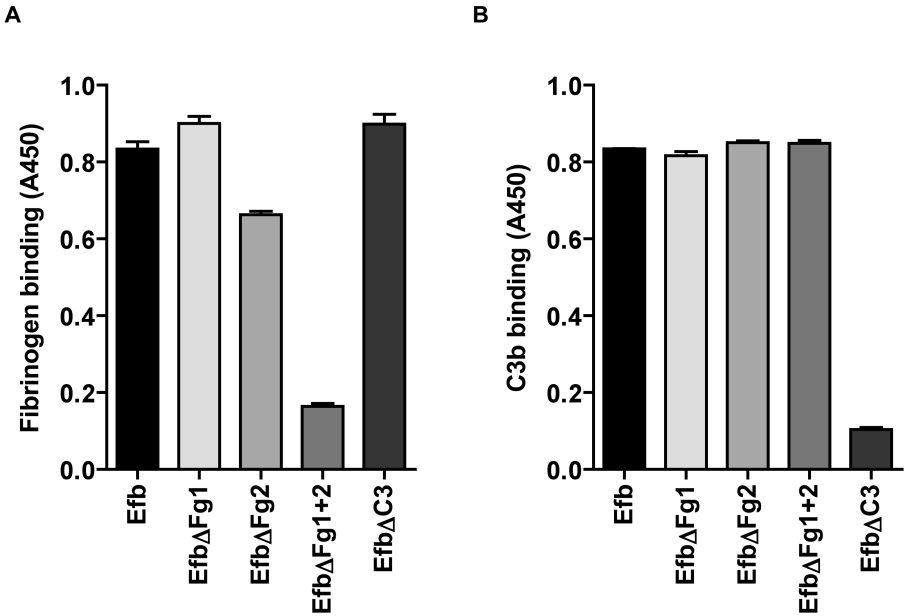

Supplement: Figure S2 — Fg- and C3b-binding characteristics of Efb mutants. ELISA experiments analyzing binding of Efb (mutants) to Fg (A) or human C3b (B). Microtiter wells, coated with 0.25 µg human Fg (A) or C3b (B), were incubated with Efb or Efb mutants (both at 6 nM). Efb binding was detected using peroxidase-conjugated rabbit anti-GST antibodies (Abcam). Data are mean ± se of two independent experiments. (PDF) [file ppat.1003816.s002.pdf]

**Supplemental figure S3**

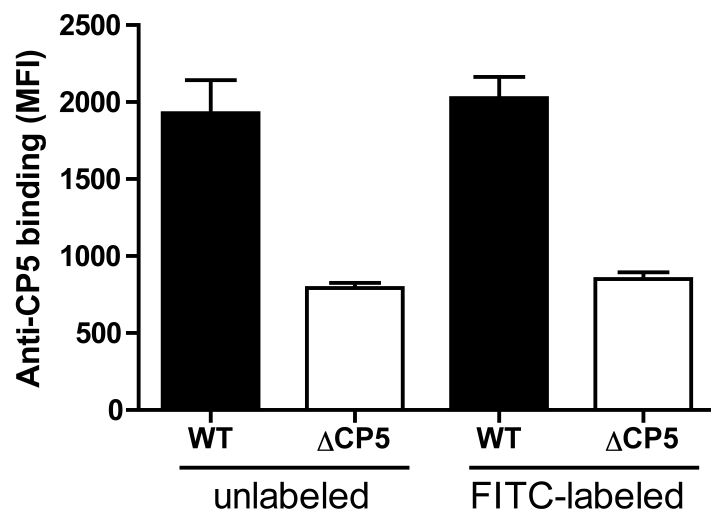

Supplement: Figure S3 — Capsule expression. (FITC-labeled) S. aureus strain Reynolds and its isogenic CP5-deficient mutant were incubated with Rabbit-anti-CP5 antibodies and PE-labeled goat-anti-rabbit antibodies. Antibody binding was quantified using flow cytometry. Data are mean ± se of two independent experiments. (PDF) [file ppat.1003816.s003.pdf]
